# Analysis on mental-insanity and cessation-of-dangerousness examinations in three Brazilian custodial institutions: a retrospective cross-sectional study

**DOI:** 10.1590/1516-3180.2020.0450.R1.22042021

**Published:** 2021-11-15

**Authors:** Tania Maria Nava Marchewka, Alvaro Nagib Atallah, Nathalia Marchewka Valente, Tamara Melnik

**Affiliations:** I PhD. Full Professor, Department of Evidence-Based Health, Universidade Federal de São Paulo (UNIFESP), São Paulo (SP), Brazil; and Researcher and Public Attorney, Public Attorney's Office of the Federal District and Territories, Brasília (DF), Brazil.; II MD, PhD. Full Professor and Head, Discipline of Emergency Medicine and Evidence-Based Medicine, Universidade Federal de São Paulo (UNIFESP), São Paulo (SP), Brazil; and Director, Cochrane Brazil, São Paulo (SP), Brazil.; III LLB. Research Assistant and Lawyer, Pontifícia Universidade Católica do Rio de Janeiro (PUC-Rio), Rio de Janeiro (RJ), Brazil.; IV PsyD, PhD, Professor, Internal Medicine and Evidence-Based Medicine, Universidade Federal de Sao Paulo (UNIFESP), São Paulo (SP), Brazil; and Researcher, Cochrane Brazil, São Paulo (SP), Brazil.

**Keywords:** Mental health, Mental health services, Psychiatric rehabilitation, Crime, Hospitals, psychiatric, Forensic psychiatry, Mental disorders, Psychiatric diagnostics, Custodial institutions, Therapeutic measures

## Abstract

**BACKGROUND:**

In Brazil, the right to healthcare and the incorporation of best scientific evidence in public health are universally guaranteed by law. However, the treatment offered to patients with mental disorders in custodial hospitals in this country has not been rigorously evaluated.

**OBJECTIVES:**

To analyze the psychiatric diagnoses and treatments implemented in three Brazilian custodial institutions.

**DESIGN AND SETTING:**

This was a retrospective, cross-sectional and descriptive study on patients held in custody in three Brazilian institutions, as judicially-determined safety measures due to their mental disorders, and the tools used in diagnoses and treatments. These institutions are in Rio de Janeiro and the Federal District.

**METHODS:**

The data from medical and judicial records that were made available were assessed regarding the diagnoses that were made and the instruments that were used.

**RESULTS:**

None of these inpatients were evaluated using validated tools, and only a few medical records presented clear descriptions of the cases. No patient with substance involvement had undergone laboratory toxicological assays. It was not possible to verify the adequacy of treatments because the procedures were inadequately described in the records.

**CONCLUSIONS:**

No standardized protocols or instruments for diagnosing mental health disorders or assessing use of psychoactive substances had been applied among the inpatients at these custodial institutions in Rio de Janeiro and the Federal District. The treatments that were prescribed to these inpatients consisted mainly of drugs.

## INTRODUCTION

The Brazilian constitution has established that healthcare is a right for all citizens and a duty of the state. Moreover, Brazilian law no. 12,401 (2011) and law no. 10,216 (2001) both give legal support for the use of the best scientific evidence in public healthcare as the basis for diagnosing and treating diseases in this country.^1–3^

In the Brazilian penal system, individuals who cannot be criminally responsible due to mental disorders cannot be penalized. Instead, security measures should be taken, and treatment should be provided. Thus, individuals with mental disorders who are considered dangerous and have committed a crime are not sent to prison in Brazil: they must comply with security measures in a custodial hospital, where patients like these should receive treatment. However, a large proportion of the inpatients at custodial hospitals for psychiatric treatment in Brazil do not receive proper medical-psychological care.^4–6^ In most cases, custodial hospitals are not integrated with the Brazilian National Health System.^5^

To the best of our knowledge, no studies have verified whether the diagnoses reported in the medical records of inpatients at these Brazilian custodial hospitals, and the treatment that these patients received, were in accordance with the recommendations and/or best scientific evidence from well-conducted clinical trials.

## OBJECTIVES

To analyze the psychiatric diagnoses and the treatments implemented in three institutions within the Brazilian Criminal Justice System through descriptive and exploratory mapping of the available reports and medical records on mental-insanity and cessation-of-dangerousness examinations.

## METHODS

### Study design and setting

This was a cross-sectional descriptive study on the diagnoses made and treatments used for inpatients, both men and women, at custodial and psychiatric treatment hospitals for individuals who had in some way conflicted with the law and required security measures (and not psychiatric patients from general hospitals and clinics). This study was conducted between March 2011 and June 2012 at an institution in Brasília, Federal District (DF), and between 2012 and 2013 at custodial hospitals in the state of Rio de Janeiro (RJ).

The present study was based on legal reports and other data included in the inpatients’ medical records. The investigation was conducted at two institutions in RJ and one in DF: the custodial and psychiatric treatment hospitals Heitor Carrilho, in the municipality of Rio de Janeiro, RJ, and Henrique Roxo, in Niterói, RJ; and the psychiatric ward of a women's penitentiary in Brasília, DF (since there are no custodial hospitals in Brasília).

All data collected during the search were obtained through personal visits to the three participating institutions, by the principal investigator and three assistants (who had previously been trained). Copies of all documents required were made. -Pre-formulated data collection forms were used in the present study to gather the variables described below. No data were obtained over the phone.

### Ethics and funding

This study was approved by the Research Ethics Committee of the Federal University of São Paulo (UNIFESP), under protocol no. 38313812.2.0000.5505, on December 18, 2014. The privacy of all participants was respected, and their identities were kept confidential. The researchers involved did not contact the participants, and the analysis was limited to assessment of documents in the medical records. Thus, there was no need for a free and informed consent statement.

The present study did not receive any form of funding from either public or private sources. The authors, who are all public employees, did not have any conflict of interest to declare regarding this study.

### Participants

All legal medical reports and data available from the medical records of inpatients at the three custodial units within the period studied were included. The participants in the present study were individuals with mental disorders in accordance with the International Classification of Diseases 10^th^ version (ICD-10).^7^ They included users of psychoactive substances who had received a diagnosis of chemical dependency and were considered not criminally responsible, in accordance with article 26 of the Brazilian penal code.^8^

Patients diagnosed with sociopathy/psychopathy were excluded because of the differentiated treatment recommended in the penal code (sole paragraph of article 26 and article 98).^8^ Such patients are considered semi-responsible for their criminal actions.

### Data collection

In the Federal District, the study was started by collecting reports and medical records that were documented in the judicial processes relating to the inpatients in the psychiatric unit.

All inpatients treated at the three institutions were identified and all documents on them that were available were evaluated (reports on mental insanity and cessation of dangerousness, reports on visits and medical consultations and, when available, the medical records containing the prescriptions for treatments).

One of the custodial and psychiatric treatment hospitals in RJ (the Heitor Carrilho Custodial and Psychiatric Treatment Hospital) was going through a process of deinstitutionalization (concluded in 2013). At the time of this study, inpatients for whom a report declaring positive cessation of dangerousness had been issued were being released. The inpatients whose families could not be located by the hospital team remained at the institution as sheltered individuals and no longer as inpatients, but still receiving assistance from the healthcare team. Thus, the Henrique Roxo Hospital became the entrance to the penal system, while the Heitor Carrilho Hospital became the exit. The entire release process is now conducted at the latter hospital, involving examinations to assess cessation of dangerousness for all inpatients who, according to the team, were in a condition that allowed them to be released. All inpatients in Rio who were included in the present study had gone through both institutions and for this reason were analyzed together.

### Variables and data analysis

The data gathered from the legal medical reports and medical records were used to define sociodemographic and family profiles, diagnoses, duration of hospitalization and therapeutic measures applied. Therapeutic projects, i.e. descriptions of treatment plans that were drawn up for individual implementation, were also included if they existed. The following data were collected:

- demographic data such as nationality, age, gender or sex of the patient, marital status, educational level, profession, address and place of birth.
- dates in which diagnostic evaluations, medical examinations and hospitalization for psychiatric treatment took place.
- mental insanity examinations that were performed: types, dates and methods (anamnesis or interview), and the instruments used for these evaluations.
- use of mental insanity examinations and structured interviews conducted by means of an instrument such as SCID (Structured Clinical Interview for DSM Disorders; New York: Biometrics Research, New York State Psychiatric Institute);^9^
- diagnoses stated in the mental insanity report and in the examination regarding cessation of dangerousness, and the security measures adopted.
- reports from the psychosocial department and social care teams.
- use of the Historical Clinical Risk Management of Violence (HCR-20) scale^10,11^ in examinations regarding cessation of dangerousness, to assess the risk of violence and security measures.
- therapeutic measures adopted: types of medications prescribed (with dosage and period) and psychotherapy.

In cases of divergence between the reports on mental insanity and cessation of dangerousness and the reports from the psychosocial sector or the medical-psychological care teams of the establishments, the findings from the report on mental insanity were given precedence.

The inpatient profile and the diagnoses and treatments received were summarized in order to facilitate comparison with the recommendations that are presented in the scientific literature.

## RESULTS

### Visits and the general situation of the files and patients

During the study period, several visits to each of the three treatment centers were needed, in order to identify cases and gain access to data. Because of the precarious situation of the infrastructure, personnel (psychiatric and psychological care, occupational therapy and social care teams) and care provided for inpatients at all three locations, many documents were missing from the inpatients’ individual files. Most of the files only included the reports from the examination for cessation of dangerousness, without the mental insanity examination. Information regarding the mental disorder that motivated hospitalization was incomplete, insufficient or absent from the reports on custodial patients. Moreover, the code of the International Classification of Diseases (ICD) was missing in many cases.

The deactivation of the Heitor Carrilho Hospital hampered the process of gathering filed data. To overcome this difficulty, hospital personnel (social assistants and part of the team of psychologists) were contacted to search for information on the patients’ situation. In 23 out of the 78 cases at Heitor Carrilho, the inpatients had grown old and had been living at the hospital for many years. Although in these cases the patients met the requirements for being released, the social care teams had been unable to contact their families to receive them, and so they remained at the institution.

In the Federal District, due to the singularity of the treatment center, it was possible to obtain mental insanity examinations and those regarding cessation of dangerousness for almost all inpatients in the feminine psychiatric unit of the local penitentiary system. However, there were no individual medical records showing whether the treatments were being properly followed.

During the period studied, 109 inpatients were identified at these three institutions. The characteristics of these individuals are described in Table 1.^12^

**Table 1:** Characteristics of inpatients in the Federal District and in Rio de Janeiro

Characteristics		Federal District (n = 39)		Rio de Janeiro (n = 70)	
**Gender**					
	Male	36	92.3%	62	88.6%
	Female	3	7.7%	8	11.4%
**Marital status**					
	Married	4	10.3%	6	8.6%
	In stable relationship	2	5.1%	-	-
	Divorced (legally)	3	7.7%	1	1.4%
	Separated	-	-	3	4.3%
	Single	27	69.2%	47	67.1%
	Widower	1	2.6%	1	1.4%
	No information	2	5.1%	12	17.1%
**Education**					
	Illiterate	1	2.6%	8	11.4%
	Middle school: partially completed*	24	61.5%	39	55.7%
	Middle school: fully completed*	3	7.7%	1	1.4%
	High school: incomplete*	-	-	2	2.9%
	High school: complete*	-	-	4	5.7%
	College/university: incomplete	1	2.6%	2	2.9%
	College/university: complete	-	-	3	4.3%
	No information	10	25.6%	11	15.7%

* Although the Brazilian educational system was modified in 1996,^12^ when the name “fundamental” started to be used to describe middle school / elementary education, and also “high school” for junior, sophomore and senior years, many medical records of the inpatients were registered with the old nomenclature (i.e. using “primeiro grau, segundo grau” etc.).

Both in the Federal District and in Rio de Janeiro, most of the inmates were men; they were either single or widowed and had low educational levels. The data on inpatients at the two hospitals in RJ were combined in the same spreadsheet, since these hospitals are under the same management.

### Diagnoses

Information on the mental disorders that led to hospitalization were incomplete, insufficient or absent in the medical records of the custodial inpatients.

Limitations found due to lack of documentation and adequate medical records are described in numbers in Table 2. This also shows in numbers the diagnostic groups affected by this negligence.

**Table 2:** Documents/medical records not found and diagnostic groups affected

Description of medical records / diagnostic groups	Federal District (n = 39)		Rio de Janeiro (n = 70)	
Mental insanity examination not found	16	41.02%	50	71.42%
Cessation of dangerousness examination not found	18	46.15%	11	15.71%
Medical or technical reports not found	13	33.33%	24	34.28%
Disease registered without ICD information	5	12.82%	26	37.14%
Mental illness	16	41%	29	41.4%
Mental illness and drug/alcohol abuse	9	23.1%	5	7.1%
Mental retardation	1	2.6%	4	5.7%
Alcohol abuse	-	-	3	4.3%
Drug abuse	1	2.6%	2	2.9%
Mental illness and mental retardation	-	-	1	1.4%
Mental and neurological illness	1	2.6%	1	1.4%
Others	2	5.1%	6	8.6%
No information	9	23.1%	19	27.1%

ICD = International Classification of Diseases.

The heterogeneity of descriptions of diagnoses in the inpatients’ records is shown in Table 3. The word “schizophrenia” was noted as a diagnosis in the records of 10 of the 45 inpatients for whom this diagnosis was reported (22%). ICD registration was present for only 7.7% of the patients in DF and for 17.1% in RJ. Moreover, different ICD codes were present in the two examinations on one patient in DF (2.6%) and one patient in RJ (1.4%).

**Table 3:** Diagnoses among inpatients in the Federal District and Rio de Janeiro

Diagnostic	Federal District (n = 16)		Rio de Janeiro (n = 29)	
Unspecified nonorganic psychosis	6	37.50%	-	-
Schizophrenia	3	18.80%	2	6.90%
Persistent delusional disorders; schizophrenia	1	6.30%	-	-
Residual schizophrenia	1	6.30%	6	20.70%
Paranoid schizophrenia	1	6.30%	6	20.70%
Unspecified psychosis not due to a substance or known physiological condition	1	6.30%	-	-
Bipolar affective disorder, manic episode with severe psychotic symptoms	1	6.30%	-	-
Delirious disorder	1	6.30%	-	-
Mental disorder (unspecified)	1	6.30%	-	-
Mental and behavioral disorders due to alcohol use	-	-	1	3.40%
Hebephrenic schizophrenia; severe schizophrenia	-	-	1	3.40%
Paranoid schizophrenia; stabilized paranoid schizophrenia	-	-	1	3.40%
Paranoid schizophrenia; borderline disorder	-	-	1	3.40%
Schizophrenia; residual schizophrenia	-	-	1	3.40%
Nonorganic psychosis	-	-	1	3.40%
Unspecified nonorganic psychosis	-	-	1	3.40%
Unspecified nonorganic psychosis; paranoid schizophrenia	-	-	1	3.40%
Chronic psychosis	-	-	1	3.40%
Residual psychosis	-	-	1	3.40%
Schizophrenic psychosis	-	-	1	3.40%
Dementia syndrome	-	-	1	3.40%
Psychotic outbreak	-	-	1	3.40%
Mood disorder	-	-	1	3.40%
Acute psychotic delusional disorder; delusional acute psychotic disorder	-	-	1	3.40%

The examinations and diagnoses regarding mental insanity and cessation of dangerousness that were conducted, with or without designation of ICD codes, are compared between Rio de Janeiro and the Federal District in Table 4. In this table, “complete diagnosis” means that presents complete information about both the diagnosis and the ICD was presented. The ICD registration was seen to be insufficient in the examinations on both mental insanity and cessation of dangerousness, in both the Federal District and Rio de Janeiro. The analysis on the medical reports and inpatients’ records was unsuccessful with regard to providing information about the ICD.

**Table 4:** Examinations and diagnoses regarding mental insanity and cessation of dangerousness, compared between Rio de Janeiro and the Federal District

		Federal District (n = 39)		Rio de Janeiro (n = 70)	
**Examinations done**					
	No examinations	2	5.1%	1	1.4%
	Mental insanity examination only	18	46.2%	13	18.6%
	Cessation of dangerousness examination only	8	20.5%	47	67.1%
	Both examinations	11	28.2%	9	12.9%
**Examinations that were diagnostic**					
	No diagnosis	9	23.1%	19	27.1%
	Diagnosis made from one examination	27	69.2%	39	55.7%
	Diagnosis made from both examinations	3	7.7%	12	17.1%
**Comparison between the ICDs in the examinations**					
	Same ICDs	2	5.1%	-	-
	Different ICDs	1	2.6%	1	1.4%
	Insufficient information	19	48.7%	19	27.1%
	No information at all	17	43.6%	50	71.4%
**Comparison between the diagnostic examinations**					
	Consistent diagnosis	2	5.1%	6	8.6%
	Inconsistent diagnosis	1	2.6%	6	8.6%
	Incomplete diagnosis	27	69.2%	39	55.7%
	No information	9	23.1%	19	27.1%
**Evaluation on the diagnosis from the mental insanity examination**					
	Complete diagnosis	20	51.3%	14	20.0%
	Incomplete diagnosis	5	12.8%	14	20.0%
	No information	14	35.9%	42	60.0%
**Evaluation on the diagnosis from the cessation of dangerousness examination**					
	Complete diagnosis	7	17.9%	7	10.0%
	Incomplete diagnosis	1	2.6%	28	40.0%
	No information	31	79.5%	35	50.0%

ICD = International Classification of Diseases.

Most diagnoses were based on either anamnesis alone or anamnesis and interviews with a family member, as shown in Table 5.

**Table 5:** How the diagnoses were made

Instruments	Federal District (n = 16)		Rio de Janeiro (n = 29)	
Anamnesis and analysis of previous medical records	4	25.0%	-	-
Anamnesis	2	12.50%	-	-
Psychometric examination, anamnesis, interview with mother and wife, analysis of previous medical records and clinical examination	1	6.30%	-	-
Anamnesis, interview with sister and analysis of previous medical and social records	1	6.30%	-	-
Anamnesis and interview with mother	1	6.30%	-	-
Anamnesis, interview with mother and analysis of previous medical records	1	6.30%	-	-
Anamnesis, interview with mother, clinical examination and analysis of previous medical records	1	6.30%	-	-
Anamnesis and interview with police officer	1	6.30%	-	-
Anamnesis, interview with sister, clinical examination, psychodiagnosis evaluation, psychometric examination and analysis of previous medical records	1	6.30%	-	-
Anamnesis, interview with father and analysis of previous medical records	1	6.30%	-	-
Anamnesis and clinical examination	1	6.30%	-	-
Anamnesis, clinical examination and analysis of previous medical records	1	6.30%	-	-
Psychiatric expertise	-	-	12	41.40%
Anamnesis and psychological examination	-	-	5	17.20%
Mental insanity examination	-	-	3	10.30%
Psychiatric medical record	-	-	1	3.40%
Ruling on the cessation of dangerousness	-	-	1	3.40%
No information	-	-	7	24.13%
**Instruments**	**Federal District (n = 9)**		**Rio de Janeiro (n = 5)**	
Anamnesis, interview with mother and sister and clinical examination	1	6.30%	-	-
Anamnesis	1	6.30%	-	-
Anamnesis, analysis of previous medical records and psychometric examination	1	6.30%	-	-
Psychometric examination, interview with mother, clinical examination and analysis of previous medical records	1	6.30%	-	-
Anamnesis, interview with mother, clinical examination, analysis of previous medical records and psychological record from IML 80/01	1	6.30%	-	-
Anamnesis, interview with brother and clinical examination	1	6.30%	-	-
Anamnesis, interview with stepfather and analysis of previous medical records	1	6.30%	-	-
Anamnesis, clinical examination, interview with mother and analysis of previous medical records	1	6.30%	-	-
Anamnesis and drug test	1	6.30%	-	-
No information	-	-	2	6.68%
Psychiatric expertise	-	-	1	3,40%
Mental insanity examination	-	-	1	3.40%
Anamnesis and psychiatric expertise	-	-	1	3.40%

IML = Medico-Legal Institute.

### Treatments

Table 6 shows the recommended treatments for these inpatients in the Federal District and Rio de Janeiro, according to their mental disorders. However, several of the reports evaluated did not state the medication that was administered to these individuals. This table presents information exactly as noted in these inpatients’ medical records.

**Table 6:** Treatments prescribed

Treatment	Federal District (n = 16)		Rio de Janeiro (n = 29)	
Hospitalization and medication	8	50.0%	-	-
Hospitalization in a place without risk of escape, medication and social service monitoring	2	12.5%	-	-
Hospitalization, medication, activities and outpatient treatment	1	6.3%	-	-
Hospitalization	1	6.3%	-	-
Hospitalization, medication and group and individual activities	1	6.3%	-	-
Hospitalization, /medication, /psychotherapy and low-sodium diet	1	6.3%	-	-
Hospitalization, medication and follow-up through “Life at Home” program	1	6.3%	-	-
Hospitalization, elimination of drugs, psychotherapy and social service monitoring	1	6.3%	-	-
No information	-	-	17	58.6%
Psychiatric treatment	-	-	2	6.9%
Outpatient treatment	-	-	2	6.9%
Interdisciplinary care aimed at building a therapeutic link and providing stability in the psychiatric setting	-	-	1	3.4%
Hospitalization in psychiatric ward and outpatient treatment	-	-	1	3.4%
Hospitalization	-	-	1	3.4%
Medication and multiprofessional treatment	-	-	1	3.4%
Medication and therapeutic treatments; patient without stable attachment to family	-	-	1	3.4%
No need for inpatient psychiatric treatment	-	-	1	3.4%
Shelter with family or in an ordinary hospital for the handicapped and disabled	-	-	1	3.4%
Medication, physical therapy and outpatient social support	-	-	1	3.4%

## DISCUSSION

### Documentation used in the study

The present study was the first exhaustive survey in Brazil on the care received by patients with mental disorders and/or chemical dependency who had been involved with conflicts with the law and who, for this reason, were inpatients at three custodial hospitals between 2011 and 2013. However, despite numerous visits to each center to obtain documentation, and contacts with administrative personnel, no comparison between the current guidelines for diagnoses and treatments and what was being practiced at these institutions was possible, simply because of the huge gaps in the records. This alone is an example of inadequacy in relation to what is recommended in international guidelines: these individuals who had committed crimes were considered to present mental disorders, but the documentation of the diagnostic process was either flawed or absent, thus indicating that there may have been a considerable amount of subjectivity in their evaluations. Judging by what was reported at these institutions, these patients had not been receiving diagnoses in accordance with standardized instruments and, therefore, their treatments could not be reviewed with regard to fulfillment of the recommendations in the literature.

This blatant negligence in the documentation of these three custodial institutions alone demonstrates some of the aspects of the precarious situation within which these patients were being treated in Brazil.^13^

### Evidence-based diagnoses

Anamnesis is only one of the elements of a psychiatric examination.^14^ A complete psychiatric examination usually includes an interview with the patient; interviews with third parties (such as family members or people who have social relationships with the individual); physical examination, with emphasis on neurological, endocrine and cardiac assessments; complementary examinations, including laboratory tests; functional tests and imaging examinations; and neuropsychological tests.^14^

Evaluators need to use their knowledge of psychopathology and, hopefully, the best scientific evidence available, to make a diagnosis.^17^ This demonstrates the importance of using structured interviews, which are objective instruments for measuring mental functions, including the risk of violence.^15–17^

The diagnosis of mental disorders may also be based on clinical data, structural neuroimaging examinations (computed tomography and magnetic resonance, etc.) and functional tests (single photon emission computed tomography, SPECT; positron emission tomography, PET; and electroencephalogram mapping, etc.). Nonetheless, psychological and neuropsychological tests are very helpful, especially for making differential diagnoses between primary psychiatric disorders (schizophrenia or primary depression, etc.) and neurological diseases.^16^

The present study showed that standardized diagnostic instruments or structured questionnaires to assess custodial inpatients were not being used at the institutions investigated. Not even family members had been interviewed, in more than half of the cases in DF and in all cases in Rio. A psychometric examination was performed on only two patients, with no description of the method or instrument applied. This means that diagnoses such as “schizophrenia”, “mental retardation”, “alcohol addiction” or others in this sample were based exclusively on the analysis of an expert examiner, who only used interviews to reach this conclusion.^16^

According to manuals such as ICD-10 and DSM,^7,18,19^ there are objective criteria that should be followed, in order to define a diagnosis of mental disorder.^20^ These include the presence of a certain number of symptoms over a defined period, to characterize the psychopathological condition. Psychiatric diagnoses and classifications of mental disorders were a matter of controversy over the course of the 20^th^ century.^16,17^

There are currently two major diagnostic systems: the one proposed by the American Psychiatric Association (APA), called the Diagnostic and Statistical Manual of Mental Disorders (DSM);^18^ and the one recommended by the World Health Organization (WHO), the Classification of Mental and Behavioral Disorders of ICD-10 (International Classification of Diseases, 10^th^ Edition). This latter system has two versions: the Clinical Descriptions and Diagnostic Guidelines (“Blue Book”) and the Diagnostic Criteria for Research (“Green Book”).^7^ The system established by the APA was created in 1980 and has been revised over the years. It is now in its fifth version.^18^ The DSM is a particularly objective instrument and is the system that best meets clinical needs, although it has not been officially adopted in Brazil. Thus, when performing a forensic analysis, Brazilian doctors must apply the classification proposed by WHO, the ICD-10, using its Chapter V, “Mental and Behavioral Disorders”.^7^ However, the ICD codes in the medical records analyzed in the present study were mostly either deficient or nonexistent. Thus, many patients may have been hospitalized without a diagnosis.

### Dangerousness tests and their accuracy

Over the past 20 years, standardized instruments have been developed to assess either dangerousness or the possibility that patients may commit violent acts under certain circumstances.^21,22^ These instruments include the following:

- Psychopathy Checklist-Revised (PCL-R): This instrument was based on the classical concept of psychopathy and contains 20 items that were chosen to assess behaviors and emotional traits that are characteristic of a psychopathic personality.^23,24^
- Barratt Impulsiveness Scale (BIS-II): This was developed to measure the three main components of impulsivity: motor component, cognitive component and absence of planning.^25,26^
- Historical Clinical Risk Management of Violence (HCR-20): This instrument was specially developed to evaluate the risk of future violent behavior in psychiatric and criminal populations. It contains historical, clinical and risk management subscales, and lists risk factors such as previous violence, young age during first violent incident, instability in relationships, work-related problems, problems regarding substance use and others.^9,10^

In the present study, there was no documentation regarding any use of any instrument for assessing levels of dangerousness (or cessation of dangerousness) among the inpatients. The HCR-20 scale,^9,10^ for example, offers the option of 10 different levels of risk of violence and could give a more objective and realistic estimate of an inpatient's situation, but it was not used in any of the cases analyzed. HCR-20 was considered to be a good predictor for violent behavior after release, in an analysis on a community of men with psychiatric disorders.^17^

This is yet another phenomenon that contributes towards perpetuation of hospitalization, as opposed to measures for resocialization and social reintegration, which are greatly emphasized in the anti-asylum movement. These individuals committed crimes but were considered not criminally responsible for their actions due to mental disorders, and they remained imprisoned even though they may have a low level of dangerousness.

In Canada, cases of individuals who are not criminally responsible on account of mental disorders (NCRMD) were recently reviewed in a set of studies conducted in three provinces.^14,16^ One of these studies^27^ also showed that there were significant heterogeneities in applying Canadian law and in the forensic procedures regarding these cases, such that individuals affected by mental disorders can be detained for longer in some provinces than in others.^16^

We consider that evaluating the accuracy of diagnostic instruments is paramount. The sensitivity and specificity of these instruments should be assessed in appropriate studies, through identification of true-positive, false-positive and true-negative cases. In this manner, appropriate conduct at diagnostic, institutional and therapeutic levels can be implemented with greater safety.

### Evidence-based treatment

The therapeutic measures recommended and those noted in the inpatient records evaluated differed greatly.^20,28–31^ The inpatient records were frequently unclear and occasionally absent. It was unclear whether some medications were being used because of their capacity to prevent psychotic events or because they promote an anxiolytic or sedative effect, thereby controlling patients’ behavior, for example. In turn, it was also unclear whether anticonvulsants were used for epileptic conditions or as mood stabilizers.^32,33^ However, considering custodial hospitals within the context of healthcare, it needs to be borne in mind that while more advanced technologies can promote better treatment results, physicians still need to be trained to use them.^4^

### Implications for practice and research

The findings from the present study indicate that there is a need for legal professionals (lawyers, attorneys and judges) to have knowledge in the field of evidence-based mental health and to be able to perform searches in the available databases.^29^ Judges in Brazil operate only on the basis of their trust in the healthcare professionals who advise them: whatever they determine is then practiced for an indeterminate time.

In the field of research, there is a clear need for better training for specialized professionals, for application of instruments that have been validated internationally to assess individuals with mental disorders. In the present study, it was seen that the professionals involved in making diagnoses and administering treatments among the inpatients apparently had not received any training or, if they had, they were not using the instruments available. Partnerships between custodial institutions and universities could assist with this problem, through identifying weaknesses in the system and proposing solutions. Universities can benefit these institutions through providing training and, in return, would find a fertile field for research within psychiatry. This idea needs to be tested through meticulously designed studies, which could include psychiatric reexaminations on patients (which was not possible in the present study).

**Figure 1:**
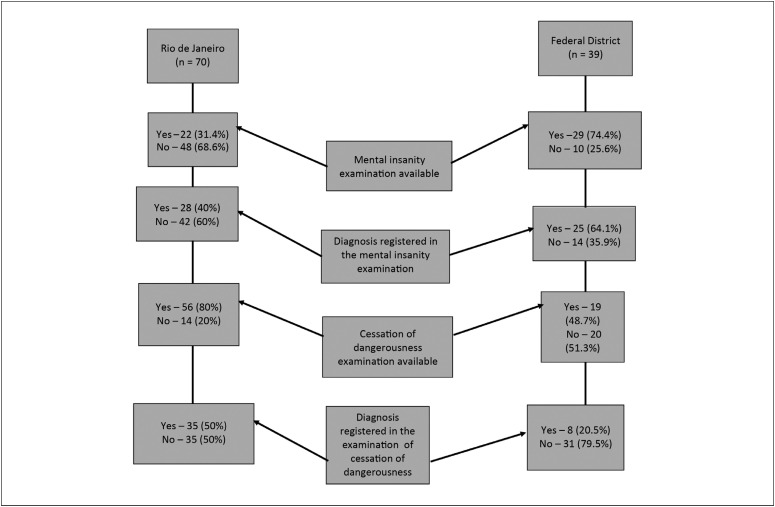
Flowchart for sample.

The flowchart (Figure 1) illustrates how it was not possible to verify the scientific credibility of the medical records due to the inadequacy of these records. It was, on the other hand, possible to verify that most of the inpatients had received a schizophrenia diagnosis, both in Rio de Janeiro and in the Federal District, albeit without further details. This was followed, in terms of frequency, by a diagnosis of use of psychoactive substances, but with no supporting drug tests or reports.

This flowchart (Figure 1) shows the distribution of information resulting from the examinations on the inpatients regarding mental insanity and cessation of dangerousness in the Federal District and Rio de Janeiro.

Thus, it became more difficult to establish a pathological profile for these inpatients who were subject to security measures. The visits made to the institutions showed, as presented in the Results section, that the lack of human resources determined the lack of individual medical records. There were absences of remedial therapists and there were some reports on cessation of dangerousness without any mental insanity examination. All of this indicates the inadequacy of the situation in relation to what is recommended by the international guidance: for instance, with regard to subjects who are considered to have a mental illness and who are therefore unaccountable before the law.

## CONCLUSIONS

This novel analysis corroborates the hypothesis that the treatments applied were not based on the current scientific evidence. Standardized instruments to assess the level of dangerousness of the inpatients at the custodial institutions were not used, either. This lack of evidence-based diagnoses makes adequate treatment impossible. There were also no standardized records regarding the recommended treatments and whether these were pharmacological.

Based on the sparse and incomplete documentation of the diagnoses presented in the medical reports, the largest proportion of the inpatients were diagnosed as having schizophrenia, with no further details, followed by the proportion with a diagnosis of use of psychoactive substances, but with no supporting toxicological screenings. This finding makes it difficult to establish a psychopathological profile for inpatients who are subject to security measures at these institutions, and to ensure quality treatment.

The lack of scientific support for these diagnoses and treatments from the best scientific evidence reveals a flaw in the integration of the fields of medicine and law. This ultimately compromises the human rights of inpatients at custodial hospitals in Brazil. These inpatients are entitled by law to the most effective and safe treatment, but the present study demonstrated that this has not been practiced.
